# Assessment of Diagnostic Yield of Cystoscopy and Computed Tomographic Urography for Urinary Tract Cancers in Patients Evaluated for Microhematuria: A Systematic Review and Meta-analysis

**DOI:** 10.1001/jamanetworkopen.2021.8409

**Published:** 2021-05-10

**Authors:** Sharon Waisbrod, Anastasios Natsos, Marian Severin Wettstein, Karim Saba, Thomas Hermanns, Christian Daniel Fankhauser, Alexander Müller

**Affiliations:** 1 Department of Urology, Spital-Limmattal, Schlieren, Switzerland; 2 Department of Urology, Kantonsspital Graubünden, Chur, Switzerland; 3 Department of Urology, University Hospital Zurich, University of Zurich, Zurich, Switzerland

## Abstract

**Importance:**

Microhematuria (MH) is a common finding that often leads to further evaluation for urinary tract cancers. There is ongoing debate about the extent to which patients with MH should be evaluated for cancer.

**Objective:**

To assess the diagnostic yield for detection of urinary tract cancers, specifically bladder cancer, upper tract urothelial carcinoma (UTUC), and kidney cell carcinoma, among patients evaluated for MH using cystoscopy and computed tomographic (CT) urography.

**Data Sources:**

MEDLINE, Scopus, and Embase were systematically searched for eligible studies published between January 1, 2009, and December 31, 2019.

**Study Selection:**

Original prospective and retrospective studies reporting the prevalence of cancer among patients evaluated for MH were eligible. Two authors independently screened the titles and abstracts to select studies that met the eligibility criteria and reached consensus about which studies to include. Among 5802 records identified, 5802 articles were screened using titles and abstracts. After exclusions, 55 full-text articles were assessed for eligibility, with 39 studies selected for systematic review.

**Data Extraction and Synthesis:**

This systematic review and meta-analysis was conducted in accordance with the Preferred Reporting Items for Systematic Reviews and Meta-analyses (PRISMA) reporting guideline. Studies were quantitatively synthesized using a random-intercept logistic regression model.

**Main Outcomes and Measures:**

The primary outcome was diagnostic yield, defined as the proportion of patients with a diagnosis of urinary tract cancer (bladder cancer, UTUC, or kidney cell carcinoma) after presentation with MH. Studies were stratified by the percentage of cystoscopy and CT urography use and by high-risk cohorts. The diagnostic yields of CT urography and cystoscopy were calculated for each cancer type.

**Results:**

A total of 30 studies comprising 24 366 patients evaluated for MH were included in the meta-analysis. The pooled diagnostic yield among all patients was 2.00% (95% CI, 1.30%-3.09%) for bladder cancer, 0.02% (95% CI, 0.0%-0.15%) for UTUC, and 0.18% (95% CI, 0.09%-0.36%) for kidney cell carcinoma. Stratification of studies that used cystoscopy and/or CT urography for 95% or more of the cohort produced diagnostic yields of 2.74% (95% CI, 1.81%-4.12%) for bladder cancer, 0.09% (95% CI, 0.01%-0.75%) for UTUC, and 0.10% (95% CI, 0.04%-0.23%) for kidney cell carcinoma. In high-risk cohorts, the diagnostic yields increased to 4.61% (95% CI, 2.34%-8.90%) for bladder cancer and 0.45% (95% CI, 0.22%-0.95%) for UTUC.

**Conclusions and Relevance:**

This study’s findings suggest that, given the low diagnostic yield of CT urography and the associated risks and costs, limiting its use to high-risk patients older than 50 years is warranted. Risk stratification, as recommended by the recent American Urology Association guidelines on MH, may be a better approach to tailor further evaluation.

## Key Points

**Question:**

What are the diagnostic yields of cystoscopy and computed tomographic urography for the detection of urinary tract cancers among patients evaluated for microhematuria?

**Findings:**

In this systematic review and meta-analysis of 30 studies comprising 24 366 patients evaluated for microhematuria, a low diagnostic yield for detecting urinary tract cancers was found for both cystoscopy and computed tomographic urography.

**Meaning:**

The study’s findings suggest that limiting the use of computed tomographic urography to patients with microhematuria who have a high risk of cancer is warranted.

## Teaser

This systematic review and meta-analysis uses data from observational studies to assess the diagnostic yields of cystoscopy and computed tomographic urography for the detection of urinary tract cancers among patients evaluated for microhematuria.

## Introduction

In recent decades, a debate has been underway regarding the optimal investigation of microhematuria (MH). There is concern that even a small amount of MH might be a sign of urinary tract cancer (UTC). However, MH is a common condition, with a prevalence ranging from 0.19% to 21.0% among adults.^1^ The high prevalence of MH leads to a considerable number of referrals for further urological workups, a situation that has substantial implications for both patients and public health. Nonetheless, consensus among national and international organizations has not yet been reached.^2^ Although the Canadian Urological Association^3^ continues to recommend that all patients with MH older than 35 years undergo evaluation using cystoscopy and that symptomatic patients be assessed using computed tomographic (CT) urography, the American Urology Association (AUA)^4^ recently updated its recommendations to limit the extent of evaluation for patients with MH. The National Board of Health and Welfare of Sweden (Socialstyrelsen) endorses no evaluation of patients with MH.^5^

More than 10 years ago, intravenous urography was replaced by CT urography as the preferred imaging modality for the upper urinary tract because of its higher accuracy in detecting upper tract urothelial carcinoma (UTUC) and kidney cell carcinoma (KCC). Nevertheless, some studies have questioned the use of CT urography as first-line imaging given the low prevalence of UTUC and concerns about radiation exposure (especially among young patients with MH) as well as the possible consequences of false-positive findings.^6,7,8,9^ The diagnostic yields of cystoscopy for detecting bladder cancer and CT urography for detecting upper UTCs among patients with MH remain uncertain. Thus, despite the plethora of published data, the ideal workup for patients with MH remains unknown. To address this issue, we conducted a systematic review of the literature published during the past decade and performed a meta-analysis of the diagnostic yields of cystoscopy and CT urography for the detection of UTCs among patients with MH to provide current data that may guide primary and secondary caregivers.

## Methods

This systematic review and meta-analysis was conducted in accordance with the Preferred Reporting Items for Systematic Reviews and Meta-analyses (PRISMA) reporting guideline.^10^ The review protocol was preregistered with the International Prospective Register of Systematic Reviews (PROSPERO) on June 10, 2019. We conducted a systematic literature search on June 30, 2019, using the Embase, Scopus, and MEDLINE databases. Our search string used combinations of synonyms and terms, including CT urography, bladder cancer, renal cancer, upper tract urothelial cancer, or urothelial cancer, coupled with microscopic hematuria or nonvisible hematuria. The detailed search strategy is shown in the eMethods in the Supplement. In addition, we manually searched the reference lists of the identified records to find additional articles. Original prospective and retrospective studies published between January 1, 2009, and December 31, 2019, that reported the prevalence of cancer among patients with MH were eligible for inclusion. We also included studies reporting the use of risk calculators or nomograms (graphic calculation devices that estimate the probability of specific events or outcomes) for the evaluation of patients with MH. We reported the areas under the curve (AUCs) published by the original authors.

No specific restrictions were applied regarding MH workup modalities or patient populations. The studies were required to report one of the following parameters: detection rate (ie, prevalence) of UTCs (bladder cancer, KCC, and UTUC) among patients with MH, type of imaging and cystoscopy used, factors associated with increased detection of cancer, or nomogram findings. Studies that were not written in English, studies with cohorts smaller than 50 patients, case reports, congressional abstracts, and reviews were excluded.

We removed duplicate articles using the close-match function in EndNote software, version X9.2 (Clarivate Analytics) and manual deduplication. Two authors (S.W. and A.N.) independently screened the titles and abstracts to select publications that met eligibility criteria and to reach a consensus about which studies to include. Data from the same study that appeared in multiple articles were only considered once in the synthesis. A data extraction sheet was developed and refined through pilot testing on 10 randomly selected eligible studies. We collected data on study design, type of evaluation, patient characteristics, prevalence of cancer and benign conditions, and risk factors. One investigator (A.N.) extracted the data, and a second investigator (S.W.) reviewed the extracted data for accuracy. All disagreements were discussed and resolved by consensus or third-party arbitration by another author (A.M.).

### Statistical Analysis

Proportions (number of cancers divided by number of patients with MH) were pooled using random-intercept logistic regression models (ie, generalized linear mixed models). The Clopper-Pearson method was used to calculate 95% CIs for individual studies. A continuity correction of 0.5 was used in studies in which no single cancer was detected. We performed meta-analyses of the diagnostic yields for detecting UTCs overall and bladder cancer, UTUC, and KCC separately, with diagnostic yield defined as the proportion of patients with a diagnosis of UTC (bladder cancer, UTUC, or KCC) after presentation with MH. We also performed subgroup analyses stratified by the percentage of CT urography and cystoscopy used (≥95% of cohort, <95% of cohort, or not reported) to examine sources of heterogeneity. We further explored subgroups based on the definition of hematuria used in each study and the prevalence of high-risk features, such as older age, male sex, and smoking history.^11,12,13,14^ High-risk cohorts were defined as groups in which the median patient age was 60 years or older, more than 50% of patients were male, and/or more than 50% of patients had a history of smoking.

The *I*^2^ statistic was used to quantify residual heterogeneity. This statistic estimates the percentage of variation across studies owing to heterogeneity rather than chance. We tested for heterogeneity using the Cochran *Q* statistic, with 2-sided *P* < .05 as the significance threshold. All analyses were performed using R software, version 4.0.0 (R Foundation for Statistical Computing).

## Results

The database search identified 5802 potentially eligible studies. Of those, 5802 articles were screened using titles and abstracts, with 55 full-text articles retrieved and assessed for eligibility. A total of 39 studies were selected for systematic review, and 30 studies comprising 24 366 patients who were evaluated for MH were included in the meta-analysis (Table and eFigure 1 in the Supplement).

**Table.** Characteristics of Included Studies

Source	Country	Study design	Total participants, No.	Participant age, mean (SD), y	No. (%)						
					Type of evaluation				Type of cancer		
					CT urography	Cystoscopy	Cytology	US	Bladder	UTUC	KCC
Abbaszadeh et al,^15^ 2009	Iran	Prospective	249	49.7 (11.8)	0	249 (100)	NR	100 (100)	7 (2.8)	NR	NR
Feifer et al,^16^ 2010	Canada	Retrospective	200	64 (NR)	12 (6.0)	200 (100)	NR	150 (75.0)	8 (4.0)	0	0
Rosser et al,^17^ 2010	US	Retrospective	85	62 (13.3)	64 (75.3)	85 (100)	85 (100)	11 (12.9)	5 (5.9)	0	1 (1.2)
Song et al,^18^ 2011	US	Retrospective	130	59 (NR)	130 (100)	NR	NR	NR	2 (1.5)	1 (0.8)	0
Cauberg et al,^19^ 2011	Netherlands	Prospective	362	56.7 (16.6)	222 (61.3)	362 (100)	362 (100)	NR	11 (3.0)	0	3 (0.8)
Ooi et al,^20^ 2011	Australia	Prospective	204	61.9 (NR)	NR	204 (100)	NR	NR	6 (2.9)	0	0
Sagnak et al,^21^ 2011	Turkey	Prospective	164	30.8 (6.4)	0	164 (100)	164 (100)	164 (100)	2 (1.2)	0	0
Cha et al,^22^ 2012	Germany and Italy	Prospective	804	65 (NR)	NR	804 (100)	804 (100)	NR	126 (15.7)	NR	NR
Karnes et al,^23^ 2012	US	Prospective	488	NR	NR	488 (100)	488 (100)	NR	15 (3.1)	NR	NR
Lokken et al,^24^ 2012	US	Retrospective	181	32.7 (6.0)	100 (55.2)	NR	NR	NR	0	0	0
Loo et al,^11^ 2013	US	Prospective	3539	NR	2212 (62.5)	3362 (95.0)	NR	956 (27.0)	27 (0.8)	0	11 (0.3)
Lee et al,^25^ 2014	South Korea	Prospective	84	NR	84 (100)	84 (100)	84 (100)	0	11 (13.1)	0	0
Lotan et al,^26^ 2014	US	Prospective	200	58 (NR)	NR	200 (100)	200 (100)	NR	5 (2.5)	NR	NR
Lisanti et al,^27^ 2014	US	Prospective	442	38.8 (NR)	442 (100)	NR	NR	NR	0	0	0
Sapre et al,^28^ 2015	Australia	Prospective	170	68 (NR)	NR	170 (100)	38 (22.4)	NR	6 (3.5)	0	0
Turkeri et al,^29^ 2014	Turkey	Prospective	303	56.6 (11.4)	34 (11.2)	303 (100)	NR	255 (84.2)	18 (5.9)	NR	NR
Bretlau et al,^30^ 2015	Denmark	Retrospective	376	NR	376 (100)	376 (100)	NR	NR	5 (1.3)	0	0
Kang et al,^31^ 2015	South Korea	Retrospective	3517	48.2 (11.0)	10 (0.3)	345 (9.8)	0	941 (26.8)	3 (0.1)	0	3 (0.1)
Bradley et al,^32^ 2016	US	Retrospective	237	67.1 (8.3)	210 (88.6)	237 (100)	NR	1 (0.4)	2 (0.8)	0	1 (0.4)
Commander,^33^ 2017	US	Retrospective	457	55.8 (NR)	457 (100)	457 (100)	NR	NR	3 (0.7)	0	0
Eisenhardt et al,^34^ 2017	Germany	Retrospective	960	58 (NR)	103 (10.7)	NR	NR	960 (100)	42 (4.4)	3 (0.3)	7 (0.7)
Elmussareh et al,^35^ 2017	Denmark	Retrospective	688	63.0 (8.3)	688 (100)	688 (100)	NR	NR	15 (2.2)	2 (0.3)	5 (0.7)
Lippman,^36^ 2017	US	Retrospective	3227	NR	NR	NR	NR	NR	20 (0.6)	0	7 (0.2)
Mace et al,^37^ 2017	US	Retrospective	84	33.0 (8.3)	84 (100)	NR	NR	0	NR	0	0
Samson et al,^38^ 2018	US	Retrospective	1049	57.0 (13.9)	1049 (100)	734 (70.0)	NR	NR	6 (0.6)	1 (0.1)	5 (0.5)
Sundelin and Jensen,^39^ 2017	Denmark	Retrospective	1305	NR	1305 (100)	1305 (100)	NR	NR	9 (0.7)	0	0
Kravchick et al,^40^ 2019	Israel	Retrospective	127	69.7 (16.9)	127 (100)	127 (100)	127 (100)	NR	9 (7.1)	7 (5.5)	NR
Janssen et al,^41^ 2018	US	Retrospective	1371	50 (18)	1371 (100)	NR	NR	NR	NR	5 (0.4)	0
Tan et al,^6^ 2018	UK	Prospective	1245	56.7 (NR)	591 (47.5)	1245 (100)	NR	758 (60.9)	33 (2.7)	0	5 (0.4)
Gonzalez et al,^42^ 2019	US	Retrospective	2118	61 (NR)	NR	2118 (100)	NR	NR	25 (1.2)	NR	NR

Abbreviations: CT, computed tomography; KCC, kidney cell carcinoma; NR, not reported; US, ultrasonography; UTUC, upper tract urothelial carcinoma.

Seven of the studies did not indicate how they defined microhematuria. Of those that provided criteria for defining microhematuria, 2 studies used a positive dipstick result as a marker, 1 study used a threshold of 5 red blood cells (RBCs) per high-power field (HPF), and the remaining 20 studies used a threshold of 3 RBCs per HPF on microscopic urinalyses of samples that were properly collected midstream according to AUA guidelines.^43^ eFigure 2 in the Supplement shows UTC prevalence stratified by the definition of MH used.

**Figure 1.**
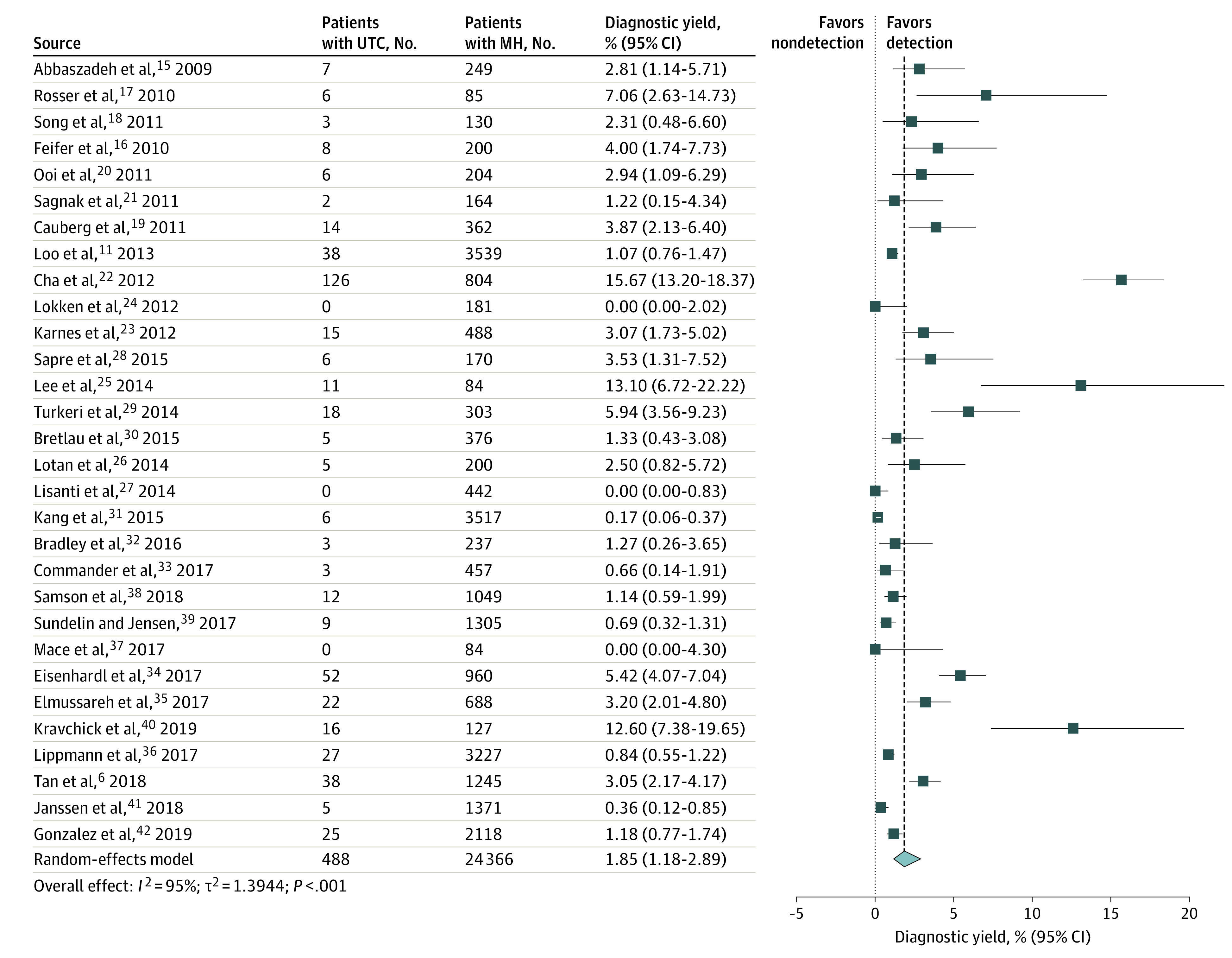
Prevalence of Urinary Tract Cancer (UTC) Across All Studies Forest plot of study-specific and pooled prevalence of UTC among patients evaluated for microhematuria (MH). Markers indicate mean values, with horizontal lines indicating 95% CIs. Diamond represents pooled mean, with point of the diamond indicating 95% CIs of the pooled mean. Additional information about the included studies is available in the Table.

A total of 488 UTCs were diagnosed among the 24 366 patients with MH included in our review. The reported prevalence of UTCs ranged from 0% to 15.67%, and the pooled detection rate among all patients was 1.85% (95% CI, 1.18%-2.89%; *I*^2^ = 95%; *P* < .001) (Figure 1). Among UTCs, bladder cancer, UTUC, and KCC comprised 420 cases (86.3%), 19 cases (3.9%), and 49 cases (9.8%), respectively.

**Figure 2.**
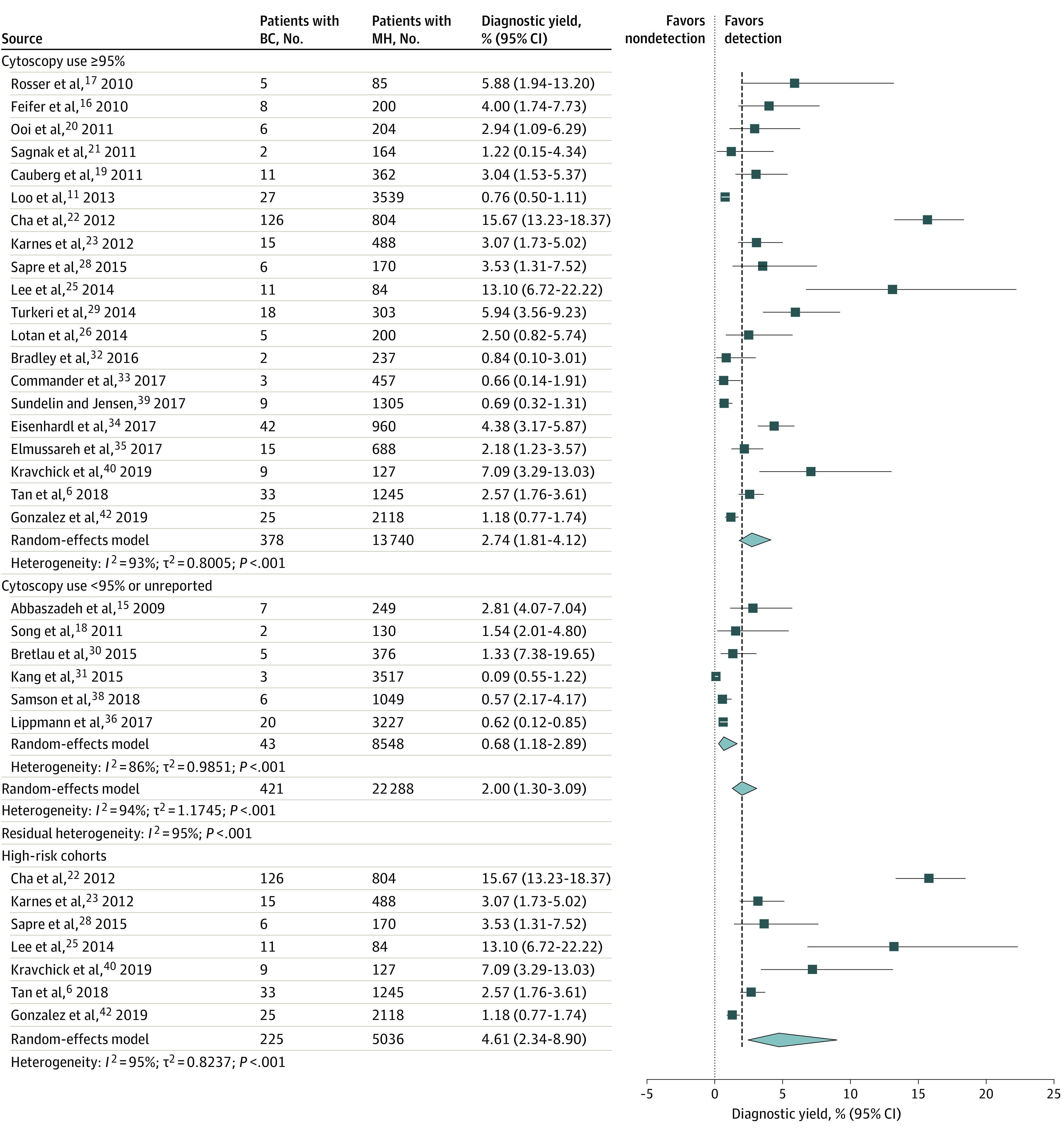
Diagnostic Yield of Cystoscopy for Bladder Cancer (BC) Forest plot of study-specific and pooled diagnostic yield of cystoscopy for BC among patients evaluated for microhematuria (MH) (stratified by use of cystoscopy in ≥95% of cohort, <95% of cohort, and high-risk cohorts). The high-risk cohort was defined as the group in which the median patient age was 60 years or older, more than 50% of patients were male, and more than 50% of patients had a history of smoking. Markers indicate mean values, with horizontal lines indicating 95% CIs. Diamonds represent pooled means, with points of the diamonds indicating 95% CIs of the pooled means. Additional information about the included studies is available in the Table.

A total of 26 studies comprising 22 228 patients with MH included evaluations of bladder cancer. Twenty studies reported the use of cystoscopy in 95% or more of the cohort. The bladder cancer detection rate ranged from 0.09% to 15.67%, and the pooled prevalence of bladder cancer was 2.00% (95% CI, 1.30%-3.09%; *I*^2^ = 94%; *P* < .001). Subgroup analyses stratified by use of cystoscopy indicated that studies in which 95% or more of the cohort underwent cystoscopy had a pooled bladder cancer detection rate of 2.74% (95% CI, 1.81%-4.12%; *I*^2^ = 93%; *P* < .001). This finding suggests a diagnostic yield of 2.74% (95% CI, 1.81%-4.12%) for the detection of MH on cystoscopy. Seven studies had high-risk cohorts comprising 5036 total participants. Subgroup analyses of these participants indicated a pooled bladder cancer detection rate of 4.61% (95% CI, 2.34%-8.90%; *I*^2^ = 95%; *P* < .001), which was almost double the diagnostic yield of cystoscopy (Figure 2).

**Figure 3.**
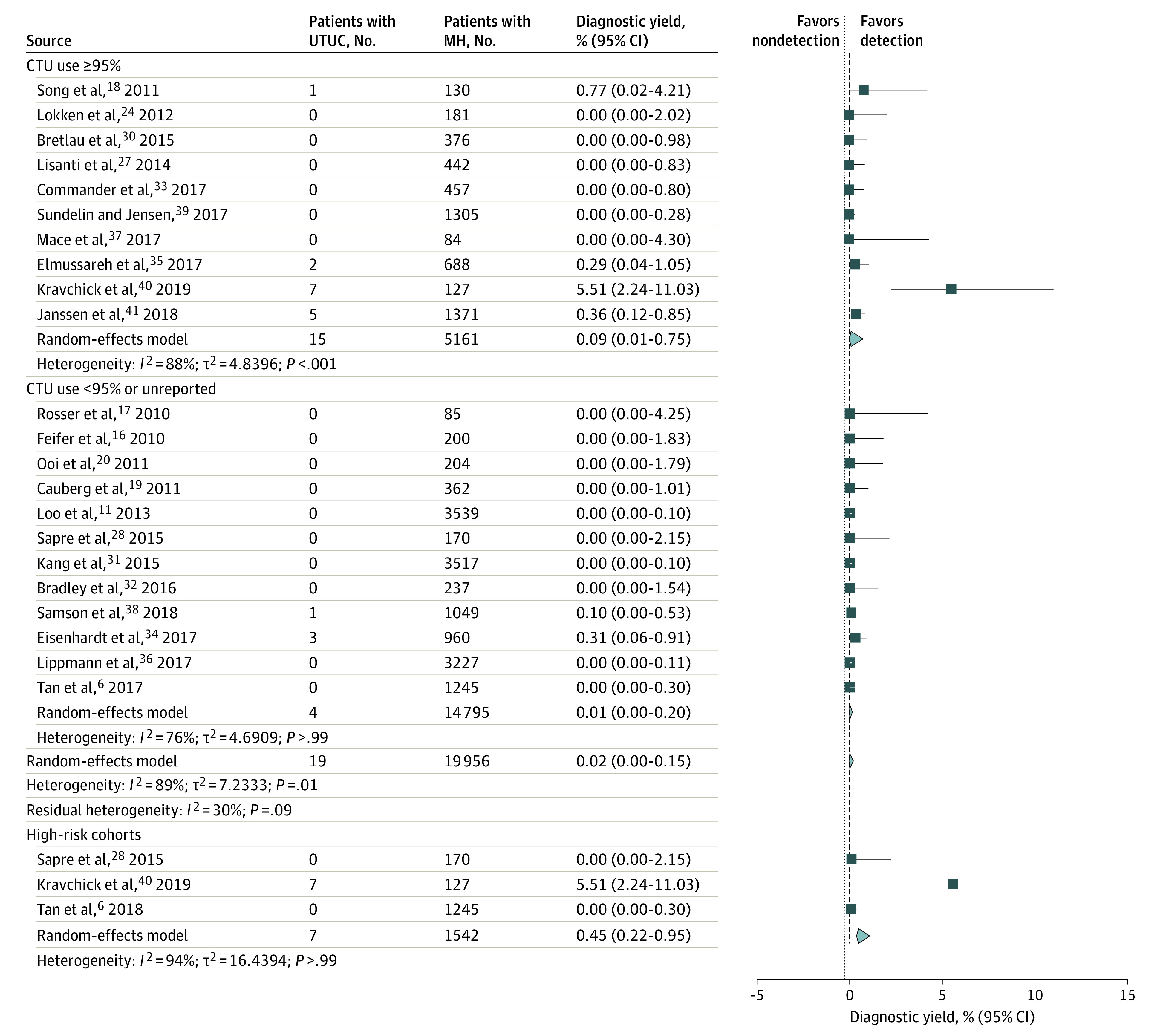
Diagnostic Yield of Computed Tomographic Urography (CTU) for Upper Tract Urothelial Carcinoma (UTUC) Forest plot of study-specific and pooled diagnostic yield of CTU for UTUC among patients evaluated for microhematuria (MH) (stratified by use of CTU in ≥95% of cohort, <95% of cohort, and high-risk cohorts). The high-risk cohort was defined as the cohort in which the median patient age was 60 years or older, more than 50% of patients were male, and more than 50% of patients had a history of smoking. Markers indicate mean values, with horizontal lines indicating 95% CIs. Diamonds represent pooled means, with points of the diamonds indicating 95% CIs of the pooled means. Additional information about the included studies is available in the Table.

**Figure 4.**
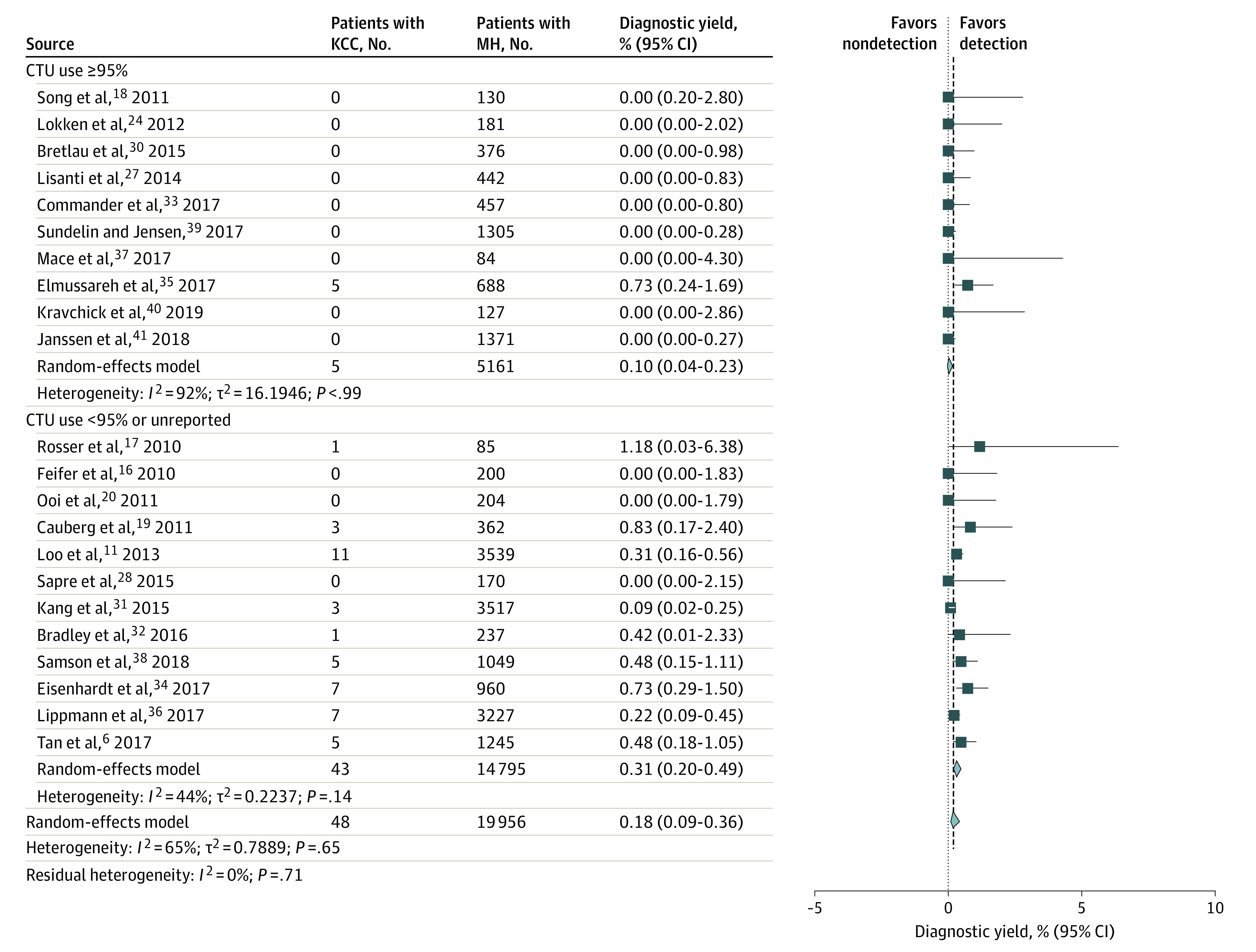
Diagnostic Yield of Computed Tomographic Urography (CTU) for Kidney Cell Carcinoma (KCC) Forest plot of study-specific and pooled diagnostic yield of CTU for KCC among patients evaluated for microhematuria (MH) (stratified by use of CTU in ≥95% of cohort and <95% of cohort). Markers indicate mean values, with horizontal lines indicating 95% CIs. Diamonds represent pooled means, with points of the diamonds indicating 95% CIs of the pooled means. Additional information about the included studies is available in the Table.

In total, 22 studies including 19 956 patients reported data on UTUC and/or KCC. Ten of those studies reported CT urography use in 95% or more of the cohort. The overall UTUC detection rate ranged from 0% to 5.51%. The pooled detection rate for UTUCs in all 22 studies was 0.02% (95% CI, 0%-0.15%; *I*^2^ = 89%; *P* = .01). After stratification, the pooled detection rate for studies using CT urography for evaluation of 95% or more of the cohort was 0.09% (95% CI, 0.01%-0.75%; *I*^2^ = 88%; *P* < .001), suggesting a diagnostic yield of 0.09% for CT urography used to detect UTUC. The stratification of studies to high-risk cohorts indicated a pooled detection rate of 0.45% (95% CI, 0.22%-0.95%; *I*^2^ = 94%; *P* > .99) (Figure 3). The KCC detection rate across the 22 studies ranged from 0% to 1.18%. The pooled prevalence of KCC in all cohorts was 0.18% (95% CI, 0.09%-0.36%; *I*^2^ = 65%; *P* = .65). Stratification to CT urography use of 95% or more indicated a detection rate of only 0.10% (95% CI, 0.04%-0.23%; *I*^2^ = 92%; *P* > .99) (Figure 4). The diagnostic yield of CT urography for both UTUC and KCC among patients with MH was 0.20% (95% CI, 0%-0.36%).

Five nomograms or risk calculators addressing risk stratification for hematuria were identified. Four of those were validated in external cohorts. All of the risk calculators included type of hematuria, age, smoking status, and sex as variables.^11,22,25,44^ Cytologic evaluation was incorporated into 2 nomograms, and the nuclear matrix protein 22 (NMP22) urinary marker was included in 1 nomogram.^22,26^ The hematuria risk score calculator indicated the greatest AUC of 0.835 (95% CI, 0.789-0.880).^45^ In comparison, the hematuria risk index calculator had an AUC of 0.809 to 0.833 (95% CIs not reported) and, in 3 cohorts, indicated a discrimination for UTC prevalence of 0.3% to 0.5% in low-risk groups and 10.8% to 11.0% in high-risk groups. No UTUC or KCC was diagnosed in the low-risk groups.^11,36^

## Discussion

To our knowledge, this systematic review and meta-analysis represents the largest and most up-to-date assessment of the diagnostic yield of cystoscopy and CT urography for the workup of patients with MH. The overall prevalence of UTCs (bladder cancer, UTUC, and KCC) among patients with MH was low, at 1.85%. Furthermore, the diagnostic yield of cystoscopy for detecting bladder cancer was 2.74%, and the diagnostic yield of CT urography for detecting both UTUC and KCC was 0.20%, reflecting the low probability of a cancer diagnosis in this population. The methods of patient selection and the extent of evaluation differed across studies, which hindered comparisons. These factors might explain the considerable heterogeneity between studies. However, the heterogeneity is also reflective of the lack of consensus across urological guidelines and highlights the current variation in clinical practice among health care professionals.^2^

Because early detection and therapy might improve the prognosis of UTC, it seems reasonable to initiate further diagnostic testing among patients with MH. However, the high prevalence of hematuria that is not associated with cancer may lead to unnecessary and potentially harmful evaluations. Our analyses suggest that indiscriminate assessment benefits only a small number of patients while exposing most patients to the potential adverse effects of further evaluations. Given that these prevalence rates represent patients referred to secondary care, it is feasible that rates in primary care are even lower. A large population-based cohort study^46^ of 772 002 patients reported a UTC detection rate of 0.68%, further highlighting the low risk of cancer in this population.

Factors associated with a higher likelihood of developing UTC among individuals with MH include older age, male sex, history of smoking, and history of visible hematuria. Seven studies recommended age thresholds between 50 and 65 years for initiating an evaluation in response to an MH diagnosis.^11,26,34,35,36,40,42^ However, a fixed age threshold is difficult to define because several studies have reported bladder cancer in patients younger than 60 years.^6,21^ One of those studies reported bladder cancer in 1.2% of patients younger than 40 years.^21^

Smoking, a known factor associated with bladder cancer and UTUC, was associated with an increased odds ratio for MH in at least 3 studies,^22,33,42^ but the cohort of the largest prospective study,^11^ which comprised 4414 patients, could not verify that smoking status was a significant factor associated with the development of cancer. There are several possible reasons for this finding. One explanation may be the lack of information about the duration of past smoking and the number of packs smoked. Another may be variations in intrinsic susceptibility to smoking-associated carcinogenesis. This issue reveals the problems involved in performing risk stratification using only 1 variable. However, nomograms, which incorporate multiple variables, can be used to increase the pretest probability for cancer detection while reducing the burden and risk associated with extensive evaluation of low-risk patients. Moreover, nomograms generally outperform clinical judgment in estimating the likelihood of an outcome, highlighting the usefulness of nomograms in everyday practice.^47^

Cystoscopy remains the criterion standard for evaluating bladder cancer.^6^ In our analysis, the diagnostic yield of cystoscopy for detecting bladder cancer was 2.74%, and this yield increased to 4.61% in high-risk cohorts. The main concerns with cystoscopy are the risk of urinary tract infections, patient discomfort, and false-positive results. Two studies reported that 39% to 40% of patients undergoing biopsies or transurethral resection of the bladder had negative results on their final histologic reports.^9,28^ These findings highlight the possible harms associated with false-positive results of cystoscopy. However, some cohorts with higher-risk characteristics had a bladder cancer prevalence as high as 15%, emphasizing the importance of performing cystoscopy among patients at risk of bladder cancer.^22,25^ A feasible solution to avoid unnecessary assessments and false-positive findings without missing the presence of bladder cancer would be to incorporate risk stratification using published risk calculators, such as the Hematuria Risk Index or the Hematuria Risk Score.^11,45^

Computed tomographic urography is considered a more accurate imaging method than intravenous urography or ultrasonography for detecting cancers of the upper urinary tract. However, our data revealed a low diagnostic yield for CT urography of only 0.09% for UTUC and 0.19% for KCC. In this analysis, no person younger than 50 years was diagnosed with UTUC. Analysis of the Surveillance, Epidemiology, and End Results database indicated that 96.3% of UTUC cases were diagnosed in patients older than 50 years, providing further data indicating the low probability of UTUC in younger patients.^48^

In addition, adverse reactions to contrast media and, more importantly, exposure to high doses of ionizing radiation up to 35 mSv are serious drawbacks to the use of CT urography.^49^ Even split-bolus protocols have radiation doses up to 15 mSv.^50^ There has been increasing concern about the association between radiation-related carcinogenesis and abdominal CT. One study estimated a risk of death from radiation-associated cancer of 0.1% in patients younger than 35 years who underwent abdominal CT.^51^ If this estimate is valid, the risk of CT urography being associated with the development of cancer among young patients may be higher than the likelihood of the evaluation detecting UTUC. Two studies that used risk models among patients with MH calculated that CT urography would be associated with more cancer-related deaths than the number of deaths due to UTUC or KCC that the modality would prevent.^8,52^ Further harm is associated with false-positive findings, which may produce consequences through either invasive diagnostic assessments, repeated imaging, or even partial or radical nephrectomy.^9,24,27,30^ Halpern et al^7^ reported that the additional diagnostic benefit of CT urography vs ultrasonography for evaluating MH would cost $6 480 484 per additional tumor detected. Therefore, the AUA updated its 2012 recommendation,^53^ in which it endorsed upper urinary tract imaging using CT urography for all patients, to recommend restricting CT urography to high-risk patients (ie, women or men aged ≥60 years, >30 pack-years, >25 RBCs per HPF on a single urinalysis, or history of gross hematuria) in its 2020 guidelines.^4^

Whether exophytic kidney tumors that have no contact with the urinary collection system are associated with MH is unknown. In a retrospective study analyzing incidental findings from abdominal CT imaging among 7365 patients, Meyer et al^54^ reported a KCC rate of 0.41%. Moreover, a meta-analysis of 16 studies reporting on 413 551 patients with abdominal ultrasonographic imaging unrelated to MH found that the prevalence of KCC was 0.1%.^55^ The similarity of these findings to ours suggests that further scrutiny of the use of CT urography for detecting KCC in patients with MH is warranted. Ultrasonography as an alternative to CT urography is now recommended in the literature as well as the updated AUA recommendations as first-line imaging for low- and intermediate-risk patients with MH.^2,4^ Ultrasonography has acceptable sensitivity for detection of KCC and a high negative predictive value for UTUC among patients with MH.^6,9^ Furthermore, ultrasonography is cost-efficient and does not expose patients to harmful radiation.^7,8^ A recent systematic review and meta-analyses by Jubber et al,^56^ which found similar detection rates, also recommended using ultrasonography as first-line imaging. Overall, upper urinary tract evaluation with CT urography imposes high costs and, in most cases, exposes patients to unnecessary harm while having a low diagnostic yield for detecting upper UTCs.

### Strengths and Limitations

This study has strengths. The main strengths are the number of patients included in the meta-analysis and the inclusion of the most recent published studies, ensuring the highest possible accuracy in reported cancer prevalence. Furthermore, this study is the first, to our knowledge, to stratify studies based on the number of patients who actually underwent CT urography and cystoscopy for MH, thereby providing a reliable assessment of the diagnostic yields of cystoscopy and CT urography.

This study also has limitations. First, the meta-analysis had considerable heterogeneity. Patient characteristics, study designs, definitions of MH, inclusion criteria, repeated testing of hematuria, exclusion of urinary tract infections, and the extent and methods of evaluation differed across studies, which may explain the different outcomes of those studies. These differences may also explain the wide gaps between the highest reported prevalence rates for bladder cancer, UTUC, and KCC (15.67%, 5.51%, and 1.18%, respectively) and the pooled prevalence rates (2.00%, 0.02%, and 0.18%, respectively). Another explanation for these gaps may be the impact of a single instance of a rare disease in small cohorts (eg, Rosser et al,^17^ Lee et al,^25^ and Kravchik et al^40^). Meta-analyses are performed precisely to compensate for such effects. Another limitation is the number of retrospective studies included because studies with this design are susceptible to variable biases in patient selection, documentation, and reporting, compromising to some extent the robustness of the overall results.

## Conclusions

The findings of this systematic review and meta-analysis revealed that a minority (1.85%) of patients with MH received a diagnosis of UTC. The maximum diagnostic yield of CT urography was 0.20% for upper UTC. The low diagnostic yield of CT urography, the risk of possible radiation-associated carcinogenesis, the high cost, and the subsequent consequences suggest that use of CT urography should be limited to high-risk patients older than 50 years. The routine use of cystoscopy to evaluate all patients with MH also seems debatable. The use of an individual risk-stratified evaluation strategy based on personal risk factors, as recommended by recent AUA guidelines,^4^ may be a better approach to assess whether further evaluation is necessary among patients with MH.

## Notes

Supplement. **eMethods.** Search String **eFigure 1.** Study Selection **eFigure 2.** Forest Plot of Study-specific and Pooled Prevalence of Urinary Tract Cancer eReferences
