# Supplementary material for: Assessment of Diagnostic Yield of Cystoscopy and Computed Tomographic Urography for Urinary Tract Cancers in Patients Evaluated for Microhematuria: A Systematic Review and Meta-analysis
Source: JAMA Netw Open. 2021 May 10;4(5):e218409. doi: 10.1001/jamanetworkopen.2021.8409 (PMC8111485; doi:10.1001/jamanetworkopen.2021.8409)
Supplement: Supplement. — eMethods. Search String eFigure 1. Study Selection eFigure 2. Forest Plot of Study-specific and Pooled Prevalence of Urinary Tract Cancer eReferences [file jamanetwopen-e218409-s001.pdf]

Supplementary Online Content

Waisbrod S, Natsos A, Wettstein MS, et al. Assessment of diagnostic yield of cystoscopy and computed tomographic urography for urinary tract cancers in patients evaluated for microhematuria: a systematic review and meta-analysis. *JAMA Netw Open*. 2021;4(5):e218409. doi:10.1001/jamanetworkopen.2021.8409

eMethods. Search String

eFigure 1. Study Selection

eFigure 2. Forest Plot of Study-specific and Pooled Prevalence of Urinary Tract Cancer

eReferences

This supplementary material has been provided by the authors to give readers additional information about their work.

© 2021 Waisbrod S et al. *JAMA Network Open*.

eMethods. Search String

bladder cancer OR transitional cell carcinoma OR urothelial cell carcinoma) AND (microhematuria OR nonvisible Hematuria) AND (CT or CT Urography OR CT AND (biomarker OR assay) (((((((hematuria OR microhematuria OR haematuria OR microhaematuria) AND (urography OR cystoscopy OR computed tomography OR pyelogram OR pyelography OR magnetic resonance imaging OR MRI OR Bladder Cancer OR cytology OR cancer OR neoplasm OR carcinoma OR malignant OR malignancy OR UTUC OR transitional cell carcinoma OR urothelial cell carcinoma OR "upper tract transitional cell carcinoma")) NOT ((newspaper article[pt] OR letter[pt] OR comment[pt] OR case reports[pt] OR review[pt] OR practice guideline[pt] OR news[pt] OR editorial[pt] OR historical article[pt] OR legal cases[pt] OR published erratum[pt] OR congresses[pt] OR subject)))

© 2021 Waisbrod S et al. *JAMA Network Open*.

eFigure 1. Study Selection

Identification

5802 records identified through database searching

5290 records remained after automatic and manual deduplication

Screening

5290 records screened by reading title and abstract

5235 records excluded after reading title/abstract:
- 1379 case reports
- 1071 letters, editorials, congress presentations
- 845 patients not evaluated for hematuria
- 573 interventional studies
- 364 reviews, guidelines
- 344 written in other language than English
- 318 evaluation for hematuria but not for urinary cancer
- 187 pediatrics
- 75 animal studies
- 48 with known cancer or prescreened
- 21 gross hematuria
- 10 Cohort less than 50 patients

Eligibility

55 full-text articles assessed for eligibility

25 excluded after reading original publication:
- 9 cancer diagnoses not reported according to type of hematuria
- 7 missing information for analyses
- 4 publications of same cohort
- 4 cohort less than 50 patients with NVH
- 1 cohort mixed with patients with bladder cancer

Included

30 studies included in qualitative synthesis

© 2021 Waisbrod S et al. *JAMA Network Open*.

eFigure 2. Forest Plot of Study-specific and Pooled Prevalence of Urinary Tract Cancer stratified according to MH-definition

Study	UTC	Nr.	%	95%−CI
**Definition by AUA guidelines**				
Abbaszadeh et al.,[16] 2009	7	249	2.81	[1.14; 5.71]
Rosser et al.,[18] 2010	6	85	7.06	[2.63; 14.73]
Song et al.,[19] 2010	3	130	2.31	[0.48; 6.60]
Feifer et al.,[17] 2010	8	200	4.00	[1.74; 7.73]
Sagnak et al.,[22] 2011	2	164	1.22	[0.15; 4.34]
Cauberg et al.,[20] 2011	14	362	3.87	[2.13; 6.40]
Loo et al.,[12] 2013	38	3539	1.07	[0.76; 1.47]
Cha et al.,[23] 2012	126	804	15.67	[13.23; 18.37]
Karnes et al.,[24] 2012	15	488	3.07	[1.73; 5.02]
Lee et al.,[26] 2014	11	84	13.10	[6.72; 22.22]
Lotan et al.,[27] 2014	5	200	2.50	[0.82; 5.74]
Lisanti et al.,[28] 2014	0	442	0.00	[0.00; 0.83]
Kang et al.,[32] 2015	6	3517	0.17	[0.06; 0.37]
Bradley et al.,[33] 2016	3	237	1.27	[0.26; 3.65]
Commander et al.,[34] 2017	3	457	0.66	[0.14; 1.91]
Samson et al.,[39] 2017	12	1049	1.14	[0.59; 1.99]
Mace et al.,[38] 2017	0	84	0.00	[0.00; 4.30]
Kravchick et al.,[41] 2017	16	127	12.60	[7.38; 19.65]
Lippmann et al.,[37] 2017	27	3227	0.84	[0.55; 1.22]
Janssen et al.,[42] 2018	5	1371	0.36	[0.12; 0.85]
Gonzalez et al.,[43] 2019	25	2118	1.18	[0.77; 1.74]
**Random effects model**	**332**	**18934**	**1.69**	**[0.94; 3.05]**
Heterogeneity: $I^2 = 96\%$, $\tau^2 = 1.7185$, $p < 0.01$				
**Definition by positive dip stick**				
Eisenhardt et al.[35], 2017	52	960	5.42	[4.07; 7.04]
Tan et al.,[6] 2018	38	1245	3.05	[2.17; 4.17]
**Random effects model**	**90**	**2205**	**4.08**	**[2.73; 6.05]**
Heterogeneity: $I^2 = 74\%$, $\tau^2 = 0.0662$, $p < 0.01$				
**Definition not reported**				
Ooi et al.,[21] 2011	6	204	2.94	[1.09; 6.29]
Lokken et al.,[25] 2012	0	181	0.00	[0.00; 2.02]
Sapre et al.,[29] 2014	6	170	3.53	[1.31; 7.52]
Turkeri et al.,[30] 2014	18	303	5.94	[3.56; 9.23]
Bretlau et al.,[31] 2014	5	376	1.33	[0.43; 3.08]
Sundelin et al.,[40] 2017	9	1305	0.69	[0.32; 1.31]
Elmussareh et al.,[36] 2017	22	688	3.20	[2.01; 4.80]
**Random effects model**	**66**	**3227**	**1.91**	**[0.97; 3.73]**
Heterogeneity: $I^2 = 84\%$, $\tau^2 = 0.6476$, $p < 0.01$				
**Random effects model**	**488**	**24366**	**1.85**	**[1.18; 2.89]**

Heterogeneity: $I^2 = 95\%$, $\tau^2 = 1.3944$, $p < 0.01$
Residual heterogeneity: $I^2 = 96\%$, $p < 0.01$

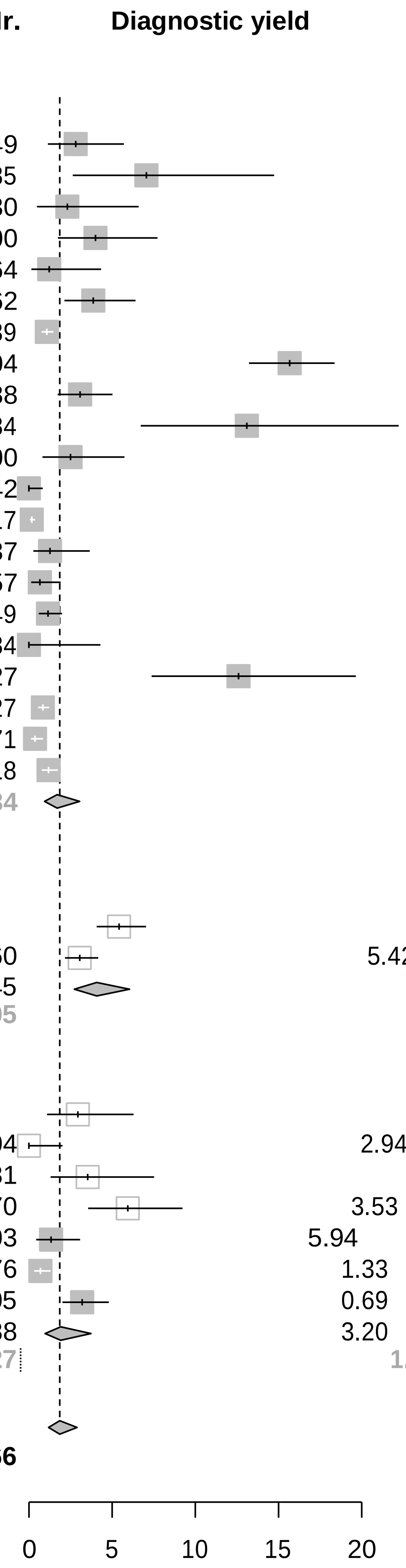

Forest plot of study-specific and pooled prevalence of UTC (%) with 95% confidence interval (CI) stratified according to MH-definition. UTC = Urinary tract cancers, MH=Microhematuria Nr. = Number of Patients with MH

© 2021 Waisbrod S et al. *JAMA Network Open*.

eReferences

6. Tan WS, Sarpong R, Khetrapal P, et al. Can Renal and Bladder Ultrasound Replace Computerized Tomography Urogram in Patients Investigated for Microscopic Hematuria? *J Urol.* 2018;200(5):973-980.

12. Loo RK, Lieberman SF, Slezak JM, et al. Stratifying risk of urinary tract malignant tumors in patients with asymptomatic microscopic hematuria. *Mayo Clin Proc.* 2013;88(2):129-138.

16. Abbaszadeh S, Taheri S, Nourbala MH. Bladder tumor in women with microscopic hematuria: an Iranian experience and a review of the literature. Adv Urol. 2009:231861.

17. Feifer AH, Steinberg J, Tanguay S, Aprikian AG, Brimo F, Kassouf W. Utility of urine cytology in the workup of asymptomatic microscopic hematuria in low-risk patients. *Urology.* 2010;75(6):1278-1282.

18. Rosser CJ, Nakamura K, Pendleton J, et al. Utility of serial urinalyses and urinary cytology in the evaluation of patients with microscopic haematuria. *West African Journal of Medicine.* 2010;29(6):384-387.

19. Song JH, Beland MD, Mayo-Smith WW. Hematuria evaluation with MDCT urography: is a contrast-enhanced phase needed when calculi are detected in the unenhanced phase? *AJR Am J Roentgenol.* 2011;197(1):W84-89.

20. Cauberg EC, Nio CY, de la Rosette JM, Laguna MP, de Reijke TM. Computed tomography-urography for upper urinary tract imaging: is it required for all patients who present with hematuria? *J Endourol.* 2011;25(11):1733-1740.

21. Ooi WL, Lee F, Wallace DMA, Hayne D. 'One stop' haematuria clinic in Fremantle Hospital, Western Australia: A report of the first 500 patients. *BJU International.* 2011;108(SUPPL.2):62-65.

22. Sagnak L, Ersoy H, Gucuk O, Ozok U, Topaloglu H. Diagnostic value of a urine-based tumor marker for screening lower urinary tract in low-risk patients with asymptomatic microscopic hematuria. *Urol Int.* 2011;87(1):35-41.

23. Cha EK, Tirsar LA, Schwentner C, et al. Accurate risk assessment of patients with asymptomatic hematuria for the presence of bladder cancer. *World J Urol.* 2012;30(6):847-852.

24. Karnes RJ, Fernandez CA, Shuber AP. A noninvasive multianalyte urine-based diagnostic assay for urothelial cancer of the bladder in the evaluation of hematuria. *Mayo Clin Proc.* 2012;87(9):835-842.

25. Lokken RP, Sadow CA, Silverman SG. Diagnostic yield of CT urography in the evaluation of young adults with hematuria. *AJR Am J Roentgenol.* 2012;198(3):609-615.

26. Lee SB, Kim HS, Kim M, Ku JH. External validation of a clinical scoring system for hematuria. *Asian Pac J Cancer Prev.* 2014;15(16):6819-6822.

© 2021 Waisbrod S et al. *JAMA Network Open.*

27. Lotan Y, Svatek RS, Krabbe LM, Xylinas E, Klatte T, Shariat SF. Prospective external validation of a bladder cancer detection model. *Journal of Urology.* 2014;192(5):1343-1348.

28. Lisanti CJ, Toffoli TJ, Stringer MT, DeWitt RM, Schwope RB. CT evaluation of the upper urinary tract in adults younger than 50 years with asymptomatic microscopic hematuria: is IV contrast enhancement needed? *AJR Am J Roentgenol.* 2014;203(3):615-619.

29. Sapre N, Hayes E, Bugeja P, Corcoran NM, Costello AJ, Anderson PD. Streamlining the assessment of haematuria: 3-year outcomes of a dedicated haematuria clinic. *ANZ journal of surgery.* 2015;85(5):334-338.

30. Turkeri L, Mangir N, Gunlusoy B, et al. Identification of patients with microscopic hematuria who are at greater risk for the presence of bladder tumors using a dedicated questionnaire and point of care urine test--a study by the members of Association of Urooncology, Turkey. *Asian Pac J Cancer Prev.* 2014;15(15):6283-6286.

31. Bretlau T, Hansen RH, Thomsen HS. CT urography and hematuria: A retrospective analysis of 771 patients undergoing CT urography over a 1-year period. *Acta Radiologica.* 2015;56(7):890-896.

32. Kang M, Lee S, Jeong SJ, et al. Characteristics and significant predictors of detecting underlying diseases in adults with asymptomatic microscopic hematuria: a large case series of a Korean population. *Int J Urol.* 2015;22(4):389-393.

33. Bradley MS, Willis-Gray MG, Amundsen CL, Siddiqui NY. Microhematuria in Postmenopausal Women: Adherence to Guidelines in a Tertiary Care Setting. *J Urol.* 2016;195(4 Pt 1):937-941.

34. Commander CW, Johnson DC, Raynor MC, et al. Detection of Upper Tract Urothelial Malignancies by Computed Tomography Urography in Patients Referred for Hematuria at a Large Tertiary Referral Center. *Urology.* 2017;102:31-37.

35. Eisenhardt A, Heinemann D, Rübben H, Heß J. Haematuria work-up in general care—A German observational study. *International Journal of Clinical Practice.* 2017;71(8).

36. Elmussareh M, Young M, Ordell Sundelin M, Bak-Ipsen CB, Graumann O, Jensen JB. Outcomes of haematuria referrals: two-year data from a single large university hospital in Denmark. *Scandinavian Journal of Urology.* 2017;51(4):282-289.

37. Lippmann QK, Slezak JM, Menefee SA, Ng CK, Whitcomb EL, Loo RK. Evaluation of microscopic hematuria and risk of urologic cancer in female patients. *Am J Obstet Gynecol.* 2017;216(2):146.e141-146.e147

38. Mace LR, Galloway TL, Ma A, et al. Diagnostic yield of CT urography in the evaluation of hematuria in young patients in a military population. *Abdom Radiol (NY).* 2017;42(7):1906-1910.

39. Samson P, Waingankar N, Shah P, Friedman D, Kavoussi L, Han J. Predictors of genitourinary malignancy in patients with asymptomatic microscopic hematuria. *Urologic Oncology: Seminars and Original Investigations.* 2018;36(1):10.e11-10.e16.

© 2021 Waisbrod S et al. *JAMA Network Open.*

40. Ordell Sundelin M, Jensen JB. Asymptomatic microscopic hematuria as a predictor of neoplasia in the urinary tract. *Scand J Urol.* 2017;51(5):373-375.

41. Kravchick S, Cherniavsky E, Verchovsky G, Peled R. Multidetector Computed Tomographic Urography (MDCTU): Its Practical Role in Diagnosis of Upper Tract Urothelial Cancer in Patients 50 years and Older with Different Types of Hematuria. *Pathology and Oncology Research.* 2019;25(1):249-254.

42. Janssen KM, Nieves-Robbins NM, Echelmeier TB, Nguyen DK, Baker KC. Could Nonenhanced Computer Tomography Suffice as the Imaging Study of Choice for the Screening of Asymptomatic Microscopic Hematuria? *Urology.* 2018;120:36-41.

43. Gonzalez AN, Lipsky MJ, Li G, et al. The Prevalence of Bladder Cancer During Cystoscopy for Asymptomatic Microscopic Hematuria. *Urology.* 2019;126:34-38.

© 2021 Waisbrod S et al. *JAMA Network Open*.
